# Nutrition Counselling Practices among General Practitioners in Croatia

**DOI:** 10.3390/ijerph14121499

**Published:** 2017-12-04

**Authors:** Albina Dumic, Ivan Miskulin, Matea Matic Licanin, Aida Mujkic, Daniela Cacic Kenjeric, Maja Miskulin

**Affiliations:** 1 Faculty of Medicine, Josip Juraj Strossmayer University of Osijek, 31 000 Osijek, Croatia; albina.dumic@gmail.com (A.D.); ivan.miskulin@mefos.hr (I.M.); matic.ma@gmail.com (M.M.L.); 2 School of Medicine, University of Zagreb, 10 000 Zagreb, Croatia; amujkic@snz.hr; 3 Faculty of Food Technology, Josip Juraj Strossmayer University of Osijek, 31 000 Osijek, Croatia; daniela.kenjeric@ptfos.hr

**Keywords:** nutrition, counselling, general practitioners, primary health care, Croatia

## Abstract

Chronic non-communicable diseases are a significant public health problem and imbalanced nutrition is one of the most significant risk factor for them. The objective of this study was to examine Croatia’s general practitioners’ nutrition counselling practice and determine the factors that influence such practice. A cross-sectional study was conducted among 444 (17.0%) randomly selected general practitioners (GPs) in Croatia from May to July 2013 via a 32-item anonymous questionnaire. Study showed that 77.0% of participants had provided nutrition counselling exclusively to patients with specific health risks; 18.7% participants had provided nutrition counselling for all patients, regardless of their individual risks, while 4.3% had not provide nutrition counselling. As the most significant stimulating factor for implementing nutrition counselling in their daily work with patients, 55.6% of the participants identified personal interest regarding nutrition and the effects it has on health. The latter factor was more frequently emphasized among female general practitioners (*p* < 0.001) and general practitioners without chronic diseases (*p* < 0.001). The most significant barrier for nutrition counselling was lack of time (81.6%). It is necessary to make additional efforts to increase the frequency of nutrition counselling provided by general practitioners in Croatia. The majority of Croatian general practitioners could increase their nutrition counselling practice in order to promote balanced nutrition and improve the overall health status of their patients.

## 1. Introduction

Chronic non-communicable diseases are a significant public health problem. Due to their lifelong nature, these diseases considerably decrease the quality of life and can lead to morbidity and premature death. Simultaneously, the diseases also represent a continuously growing burden on the healthcare system and the economy in general. As the leading cause of death globally, chronic non-communicable diseases were responsible for 38 million (68%) of the world’s 56 million deaths in 2012. Chronic non-communicable diseases are connected to the same risk factors, among which imbalanced nutrition is one of the most significant. At the same time, the development of chronic non-communicable diseases are mostly preventable; most primary prevention strategies are aimed at modifying lifestyle and individual risk factors [1].

General practitioners (GPs) are ideally placed within the healthcare system to provide their patients with adequate counselling that can help in the prevention of the chronic non-communicable diseases [2,3,4]. Doctors at the primary level of health care can provide counselling in their offices. Such counselling represents an opportunity to further influence each patient's specific preventive behaviors, through evaluating the patient’s dietary plan, which is related to excess weight, obesity, and a variety of other chronic non-communicable diseases [5].

Around 70% of adults visit their GP at least once a year and 90–95% adults visit in a three-year period, confirming the important position of the GP in the prevention of chronic non-communicable diseases [6,7]. In addition, studies have shown that nutritional effects on health is the main topic in 14–28% of the total patient counselling done by doctors in the field of family medicine [7]. Although estimates from individual experts in the field of nutrition vary, more than 25% of patient visits to GPs are thought to be due to some health disorder related to improper nutrition [8]. This clearly indicates the potential for preventive action that lies with GPs in every field, but mostly in the field of nutrition counselling, which unfortunately has not been recognized, so preventative activities are rarely practiced [2,6,7,9,10,11].

The objective of this study was to examine the nutrition counselling practices of Croatia’s general practitioners and determine the factors that influence these practices.

## 2. Materials and Methods

### 2.1. Participants

This cross-sectional study was conducted from 1 May to 31 July 2013 among doctors who work in family medicine offices in Croatia and are entered in the register of GPs, which is managed by the National Institute of Public Health. The study was approved by the Ethical Board of the Faculty of Medicine Osijek, Croatia (Ethical Approval Code: 2158-61-07-12-35).

Among the doctors who work in family medicine offices in Croatia, and are registered in the register of GPs led by the National Institute for Public Health, 800 potential participants were chosen by random selection and were sent an anonymous questionnaire via post. Along with the questionnaire, each GP, a potential participant, had received an explanation of the study and an informed consent form. After reading about the aim and purpose of the study, all of the participants were asked to sign the consent form before answering the questionnaire. Aside from the previously mentioned documents, the informed consent form, study explanation for the participants, and anonymous questionnaire, each doctor received two envelopes which contained the address of the lead author. They were asked to put the signed informed consent form in one envelope, and the answered questionnaire in the other envelope and then send them to the lead author. Thereby the anonymity of the study was ensured and by no means could the personal data, specifically the name and surname, of the participants be connected to the answers provided in the questionnaire. The answered questionnaires that arrived via post were assigned codes and the data provided within the questionnaires were later analyzed using that code. Out of the 800 potential participants, 444 responses were received, which is a response rate of 55.5%. The study included 17.0% of doctors who work in family medicine offices in Croatia and the study participant sample was representative for this population of Croatian doctors. 

### 2.2. Measures

The study was conducted through an anonymous self-administered questionnaire composed of 32 questions divided into four categories. The first category of questions referred to the characteristics of the participants, including age, gender, education, length of service, additional education in the field of nutritional science, and ailments from chronic non-communicable diseases where improper nutrition poses as a risk factor. The second category of questions referred to the GP’s knowledge of the field of nutritional science. The third category of questions involved the opinions the GPs had about nutrition and counselling about nutrition. The fourth category referred to the nutrition counselling implementation performed by GPs in their daily work with patients and also asked about the factors that motivate or even discourage doctors in their attempt to implement nutrition counselling in their daily work with patients. The questionnaire used in this study was previously validated with a smaller group of participants in 2012.

Within this study, questions about the participants’ characteristics and questions about the nutrition counselling implementation were analyzed.

### 2.3. Data Analysis

For the description of the frequency distributions of the examined variables, descriptive statistical methods were used. All of the variables were tested for normality distribution using the Kolmogorov-Smirnov test. The middle values of the continuous variables were expressed as a median and a range of variables that were not distributed normally. Nominal indicators were displayed by the frequency distribution according to groups and participation. For determining the difference between two independent sample proportions, the χ²-test and Fisher exact test were used. For the mean grade point average of the obtained results, a *p* < 0.05 level of significance was selected. When analyzing data, source programs for data bases and the statistical package Statistica for Windows 2005 (version 7.1, StatSoft Inc., Tulsa, OK, USA) were used.

## 3. Results

A total of 444 participants were included in the study, and the respondents were 81.3% women and 18.7% men. The average age of all the participants was 50 years old, ranging from 25 to 67. Among all the participants, 67.3% were specialists in family medicine and 32.7% were doctors who had licenses for an independent practice, but without a finished specialization in the field of family medicine. According to the length of service, 65.1% of the participants had 15 or more years of service and 34.9% had 0–14 years of work experience. Among the participants 9.5% had completed additional educational programs in the field of nutritional science and 30.6% had suffered from some chronic non-communicable disease with improper nutrition posing as a risk factor.

Throughout their daily work, 77.0% of participants provide nutrition counselling only for patients considered at risk regarding their dietary habits and/or body mass index, 18.7% of the participants always conduct nutrition counselling with their patients regardless of their individual health risks, whereas 4.3% of the participants do not conduct nutrition counselling at all. Nutrition counselling for every patient, regardless of his or her individual risks, was conducted more often by women (86.7%), specialists in family medicine (68.7%), participants with 15 or more years of work experience (71.1%), participants who had not acquired additional education in the field of nutritional science (73.5%), and by participants who do not suffer from some chronic non-communicable disease with improper nutrition posing as a risk factor (74.7%). Also, participants who provided nutrition counselling only for patients considered at risk regarding their dietary habits and/or body mass index were more often women (79.8%); specialists in family medicine (67.8%); participants with 15 or more years of work experience (63.7%); participants who had not acquired additional education in the field of nutritional science (94.2%); and participants who do not suffer from some chronic non-communicable disease with improper nutrition posing as a risk factor (67.0%).

**Figure 1:**
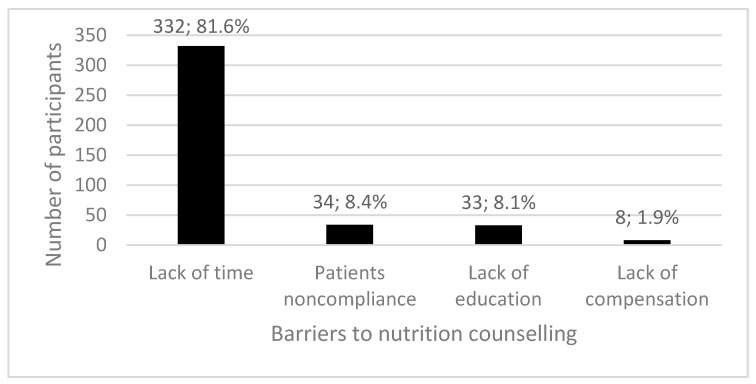
Barriers to nutrition counselling among Croatia’s general practitioners.

As the most significant stimulating factor for implementing nutrition counselling in their daily work with patients, 55.6% of the participants identified personal interest regarding nutrition and the effects it has on health, and 44.4% of the participants identified the acquired additional education in the field of nutritional science. Of the participants, 91.7% encountered certain barriers when conducting nutrition counselling, and 8.3% of the participants conduct counselling without any barriers. Lack of time was identified as the most frequent barrier among 81.6% of participants that encounter barriers during nutrition counselling, while other barriers were rarely encountered (Figure 1).

Table 1 displays the participants who conduct nutrition counselling for every patient without experiencing barriers and those who experience some barriers based on their demographic characteristics.

Table 2 displays participants who conducted nutrition counselling, without barriers, for patients considered at risk in regards to their dietary habits and/or body index mass and those who encounter barriers, according to their demographic characteristics.

Table 3 displays the stimulating factors for nutrition counselling among Croatia’s general practitioners according to their demographic characteristics. Women (*p* < 0.001) and GPs without chronic diseases (*p* < 0.001) more often identify personal interest in nutrition and the effects it has on health as a stimulating factor for nutrition counselling.

Table 4 shows the barriers in conducting nutrition counselling among Croatia’s general practitioners according to their demographic characteristics. Specialists in family medicine (*p* = 0.017), GPs with 15 or more years of work experience (*p* = 0.045), GPs with no additional education about nutrition (*p* = 0.001), and GPs without chronic diseases (*p* = 0.049) more often identify lack of time as a leading barrier to conducting nutrition counselling.

## 4. Discussion

The present study showed that in their daily work, 18.7% of Croatia’s GPs always conduct nutrition counselling with their patients, regardless of their individual health risks, whereas 77.0% conduct nutrition counselling only for patients considered at risk regarding their dietary habits and/or body mass index. The results related to the counselling of each patient are quite similar to the results of the study conducted among Canadian GPs in 2006 in one of the 10 Canadian provinces that showed that 19.1% of doctors provided their patients with nutrition counselling [10]. Moreover, the results are worse than the results of a much larger study conducted in Canada the same year, based on four out of 10 Canadian provinces, which showed that 38.7% of Canadian GPs provided nutrition counselling for their patients [2]. Regarding nutrition counselling for patients with specific health risks, a study conducted among German doctors showed that during their patients’ preventive examinations, 78% of doctors provided nutrition advice to at-risk people due to their body mass index [12]. On the other hand, a study conducted among GPs in Australia showed that less than a third of obese patients were provided nutrition counselling aimed at reducing weight, whereas around a third of patients suffering from high blood pressure were provided nutrition counselling aimed at reducing salt intake [13]. A mentioned study in Australia, also, showed that obese patients suffering from high blood pressure were more often provided with nutritional advice by their GP, as opposed to obese patients who did not suffer from high blood pressure. A study among Australian doctors, as well as other studies, stressed that some GPs are more likely to provide nutrition counselling to individuals with specific health risks, especially if they suffer from more than one risk [13,14]. This study shows a greater ratio of Croatia’s GPs provide nutritional advice to patients with some risks, as opposed to the ratio of those who provide nutrition counselling for all patients, regardless of their individual risks. Despite the fact that 77.0% of Croatia’s GPs always provide nutrition counselling for patients considered at risk with regards to dietary habits and/or body mass index, there is room for further improvement, especially in Croatia where excess body weight and obesity are significant public health problems [15]. Studies have alarmingly shown that the percentage of obese and overweight people continues to grow and how these described trends impair the health and life expectancy of present and future generations in Croatia [15,16]. Therefore, nutrition counselling is crucial for overweight or obese people in Croatia. It is important to be sensitive to the several studies that have shown that doctors respect obese individuals less, since they believe that these patients lack motivation for body weight regulation, ultimately affecting the manner in which GPs communicate with such individuals [17]. Further study among GPs have demonstrated that they express less empathy, worry, and partnership support for obese patients as opposed to patients with normal body weight [18]. The impaired communication between obese patients and their GPs could therefore be one of the key factors that should be taken into account when examining the identified ratio of Croatian GPs who provide nutrition counselling for patients at risk. Moreover, based on the restricted study conducted among GPs in Croatia in 2003, only 14% of GPs considered improper nutrition as an important health public problem. In addition, 66% of GPs see their primary role solely in individual work with patients regarding nutrition that have health problems due to excess body weight (tertiary prevention), and much less discern their role in primary and secondary prevention [19]. These are important additional factors that have led to the determined levels of nutrition counselling among Croatia’s GPs and have also led to a higher percentage of those who provide counselling solely to individuals with specific health risks and hardships.

When examining the demographic characteristics of Croatia’s GPs that provide nutrition counselling for every patient regardless of his or her individual risks and those that solely provide nutrition counselling for patients with certain health risks, the same pattern was discovered. This pattern shows that counselling is more often conducted by women, specialists in family medicine, doctors with 15 or more years of work experience, doctors who had not acquired additional education in the field of nutritional science, and by doctors who do not suffer from some chronic non-communicable disease with improper nutrition posing as a risk factor. In the latter and previous study, specialists in family medicine provided nutrition counselling more often in Croatia, and the same was also demonstrated in a study among American doctors [19,20,21]. In addition, studies have shown that counselling was conducted more often by women and older doctors, specifically, and doctors with more years of work experience [20,21,22], as was shown in this study. A study conducted among American doctors also showed that doctors who completed additional educational programs in the field of nutritional science and doctors who experienced weight issues conducted nutrition counselling more often [22], but this was not demonstrated in this study. The latter can be explained by the fact that, for a majority (55.6%) of Croatia’s GPs, the most significant stimulating factor for implementing nutrition counselling was personal interest regarding nutrition and its effects on health and for only 44.4% of them, the completed additional education in the field of nutritional science inspired implementing nutritional counselling. The fact that women and doctors who do not suffer from some chronic non-communicable disease with improper nutrition posing as a risk factor more often identified personal interest regarding nutrition, and the effects it has on health as a stimulating factor for nutrition counselling, can be interpreted by considering that women around the age of 50 make up the majority of GPs in Croatia and by the fact that women of that age in Croatia suffer less from the mentioned chronic non-communicable diseases.

When examining the barriers that Croatia’s GPs encounter during nutrition counselling, participants most frequently identified the lack of time (81.6%), patient non-compliance (8.4%), a lack of education in the subject matter field (8.1%), and finally, lack of compensation (1.9%). These results are similar to those reported elsewhere in the world, but certain proportion differences exist that are attributed to the previously mentioned patient, and possible barriers encountered during nutrition counselling [8,10,23,24,25,26,27]. These results differ from the results of an earlier study among Croatia’s GPs where the most significant barrier, which was four times as frequent from the determined proportions in this study, was patient non-compliance [19]. A lack of time was identified as a barrier three time less during nutrition counselling. In the latter study, a higher percentage (22.1%) of participants identified the lack of education in the subject matter field as a barrier during counselling, and a more significant percentage (24.6%) identified a lack of knowledge in terms of conducting such counselling. This was not found in this study [19]. We concluded that in a 10-year period from 2003 to 2013, the daily practice of GPs in Croatia has undergone changes in terms of the amount of received workload, leading to a lack of time, which now poses as a key barrier in conducting nutrition counselling. On the other hand, based on the compared results, a positive trend is evident among patients who, empowered by the accessible information in relation to nutritional effects on health, have become much more cooperative and prepared for a partnership with their GPs with the goal of improving one’s own health. Finally, it appears that the medical training in Croatia has been able to respond to the needs for additional knowledge dissemination about nutritional effects on health by improving the curriculum and providing permanent training for doctors, as recommended in other studies [28,29]. Because of the implemented additional training for doctors about nutritional effects on health, the percentage of doctors who identified a lack of education in the subject field as a main barrier in conducting nutrition counselling dropped by 14.0% in the observed 10-year period, but there is still room for further improvement. The fact that specialists in family medicine more often encounter barriers such as lack of time during nutrition counselling is most probably related to the fact that specialists in family medicine provide nutrition counselling more often to every patient, including those with some health risks, as opposed to doctors with a license for independent practice, without a finished specialization, working as GPs. Furthermore, the lack of time as a barrier for nutrition counselling was more often identified by the GPs with 15 or more years of work experience, probably related to the fact that those doctors have larger practices with more patients than the younger ones. As only 9.5% of Croatia’s GPs have completed additional educational programs in the field of nutritional science and the majority of them (69.4%) do not suffer from some chronic non-communicable disease with improper nutrition posing as a risk factor, it is not surprising that these doctors more often identified lack of time as a barrier for nutrition counselling.

This study has several limitations. Firstly, the data related to nutrition counselling among GPs in Croatia was based on the doctor’s reported statements about counselling implementation. It is socially and professionally acceptable to regularly counsel one’s own patients about nutrition, the identified percentage of those who conduct counselling may be overestimated or inflated. Consequently, in future studies related to doctors who conduct nutrition counselling every day, for instance, questionnaires should be simultaneously provided to both doctors and patients. Secondly, questions related to nutrition counselling implementation did not question the types of counselling and whether counselling methods used were appropriate for the specific needs of the individuals experiencing health risks. This should be taken into consideration when conducting similar studies in the future since some types of counselling are more successful in changing poor dietary habits than others [30]. In addition, a previous study in Croatia showed that a significant percentage of GPs pointed to a lack of familiarity concerning the conduction of nutrition counselling as one of the major barriers during counselling [19]. Therefore, future studies should evaluate the types of nutrition counselling that GPs conduct in Croatia and investigate in detail the circumstances in which these types of counselling occur. This study has also shown that most counselling (77.0%) is conducted with individuals that experience specific health risks and the overall counselling is related to treatment. Therefore, physicians should be encouraged to provide information about a balanced diet to every patient thus guiding them toward action in terms of primary disease prevention, which is an effective approach among the population of healthy adults, at the level of primary healthcare [31].

Despite studies that have shown how the percentage of obese and overweight people continues to grow and how these trends further impair the health and life expectancy of present and future generations in Croatia [15,16], this study discovered that, on a daily basis, only 18.7% of GPs in Croatia always conduct nutrition counselling with their patients, regardless of their individual health risks. A short educational intervention conducted by GPs can encourage multiple dietary changes, which would lower the participant’s body mass index and consequently reduce the risks of suffering from chronic non-communicable diseases among the population of healthy adults [31]. In addition, within the Croatian healthcare system, as a freely-selected GP is the person through which insured individuals access all health services [19], doctors need to increase the frequency of nutrition counselling practices provided to each patient. Moreover, as a selected GP in Croatia usually treats the entire family, this can be an additional supporting factor for providing continual counselling and managing a whole family-based counselling approach. This is an important success pre-determiner of public health intervention, since dietary habits are acquired in early childhood, within the family circle, and affect the health of a person in their adult age [32].

## 5. Conclusions

The majority of Croatian GPs could conduct nutrition counselling more often and promote a balanced diet among their patients, as it would improve their patient’s health. In order to achieve this goal, additional efforts and encouraging GPs in Croatia to conduct nutrition counselling is required. Incentives should be provided for all participants who are dealing with this issue, from the Faculty of Medicine that educates future doctors, Ministry of Health and Croatian National Institute for Health Insurance, which evaluates the work of a doctor, and finally from medical professional associations which conduct continual medical training programs. In addition, a health policy aimed at primary prevention and health promotion, should definitely be a part of these efforts to more successfully implement nutrition counselling at the primary level of health care in Croatia.

## Figures and Tables

**Table 1:** General practitioners in Croatia who provide nutrition counselling for every patient, with or without barriers, according to their demographic characteristics.

Participant Characteristics	Number of Participants (%)			*p* *
	Counselling without Barriers	Counselling with Barriers	Overall	
**Gender**				
Male	2 (8.7)	9 (15.0)	11 (13.3)	0.507 *
Female	21 (91.3)	51 (85.0)	72 (86.7)	
**Education**				
Doctors with a license for independent practice without a finished specialization	7 (30.4)	19 (31.7)	26 (31.3)	1.000 ^#^
Specialist in family medicine	16 (69.6)	41 (68.3)	57 (68.7)	
**Length of Service (Years)**				
0–14	5 (21.7)	19 (31.7)	24 (28.9)	0.429 ^#^
15 or more	18 (78.3)	41 (68.3)	59 (71.1)	
**Additional education about nutrition**				
Yes	5 (21.7)	17 (28.3)	22 (26.5)	0.593 ^#^
No	18 (78.3)	43 (71.7)	61 (73.5)	
**Ailment from Chronic Diseases**				
Yes	4 (17.4)	17 (28.3)	21 (25.3)	0.403 ^#^
No	19 (82.6)	43 (71.7)	62 (74.7)	
Overall	23 (100)	60 (100)	83 (100)	

* Fisher exact test; ^#^ χ^2^ test.

**Table 2:** General practitioners in Croatia who provide nutrition counselling, without or with some barriers, for patients experiencing some health risks according to their demographic characteristics.

Participant Characteristics	Number of Participants (%)			*p* *
	Counselling with at Risk Patients without Barriers	Counselling a with at Risk Patients with Barriers	Overall	
**Gender**				
Male	1 (10.0)	68 (20.5)	69 (20.2)	0.488
Female	9 (90.0)	264 (79.5)	273 (79.8)	
**Education**				
Doctors with a license for independent practice without a finished specialization	3 (30.0)	107 (32.2)	110 (32.2)	1.000
Specialist in family medicine	7 (70.0)	225 (67.8)	232 (67.8)	
**Length of Service (Years)**				
0–14	1 (10.0)	123 (37.0)	124 (36.3)	0.101
15 or more	9 (90.0)	209 (63.0)	218 (63.7)	
**Additional Education about Nutrition**				
Yes	2 (20.0)	18 (5.4)	20 (5.8)	0.110
No	8 (80.0)	314 (94.6)	322 (94.2)	
**Ailment from Chronic Diseases**				
Yes	2 (20.0)	111 (33.4)	113 (33.0)	0.507
No	8 (80.0)	221 (66.6)	229 (67.0)	
Overall	10 (100)	332 (100)	342 (100)	

* Fisher exact test.

**Table 3:** Factors promoting nutrition counselling among Croatia’s general practitioners according to their demographic characteristics.

Participant Characteristics	Number of Participants (%)			*p* ^#^
	Personal Interest Related to Nutrition and the Effect of Nutrition on Health	Acquired Additional Education in the Field of Nutritional Science	Overall	
**Gender**				
Male	31 (12.6)	52 (26.4)	83 (18.7)	<0.001
Female	216 (87.4)	145 (73.6)	361 (81.3)	
**Education**				
Doctors with a license for independent practice without a finished specialization	83 (33.6)	62 (31.5)	145 (32.7)	0.684
Specialist in family medicine	164 (66.4)	135 (68.5)	299 (67.3)	
**Length of Service (Years)**				
0–14	91 (36.8)	64 (32.5)	155 (34.9)	0.368
15 or more	156 (63.2)	133 (67.6)	289 (65.1)	
**Additional Nutrition Education**				
Yes	27 (10.9)	15 (7.6)	42 (9.5)	0.257
No	220 (89.1)	182 (92.4)	402 (90.5)	
**Ailment from Chronic Diseases**				
Yes	58 (23.5)	78 (39.6)	136 (30.6)	<0.001
No	189 (76.5)	119 (60.4)	308 (69.4)	
Overall	247 (100)	197 (100)	444 (100)	

^#^ χ^2^ test.

**Table 4:** Barriers for nutrition counselling among Croatia’s general practitioners according to their demographic characteristics.

Participant Characteristics	Number of Participants (%)				*p*
	No Barriers	Lack of Time	Other Barriers	Overall	
**Gender**					
Male	3 (8.1)	62 (18.7)	18 (24.0)	83 (18.7)	0.119 ^#^
Female	34 (91.9)	270 (81.3)	57 (76.0)	361 (81.3)	
**Education**					
Doctors with a license for independent practice without a finished specialization	12 (32.4)	98 (29.5)	35 (46.7)	145 (32.7)	0.017 ^#^
Specialist in family medicine	25 (67.6)	234 (70.5)	40 (53.3)	299 (67.3)	
**Length of Service (Years)**					
0–14	6 (16.2)	121 (36.4)	28 (37.3)	155 (34.9)	0.045 ^#^
15 or more	31 (83.8)	211 (63.6)	47 (62.7)	289 (65.1)	
**Additional Nutrition Education**					
Yes	7 (18.9)	21 (6.3)	14 (18.7)	42 (9.5)	0.001 *
No	30 (81.1)	311 (93.7)	61 (81.3)	402 (90.5)	
**Ailment from Chronic Diseases**					
Yes	7 (18.9)	112 (33.7)	17 (22.7)	136 (30.6)	0.049 ^#^
No	30 (81.1)	220 (66.3)	58 (77.3)	308 (69.4)	
Overall	37 (100)	332 (100)	75 (100)	444 (100)	

* Fisher exact test; ^#^ χ^2^ test.
